# Mitotic Evolution of *Plasmodium falciparum* Shows a Stable Core Genome but Recombination in Antigen Families

**DOI:** 10.1371/journal.pgen.1003293

**Published:** 2013-02-07

**Authors:** Selina E. R. Bopp, Micah J. Manary, A. Taylor Bright, Geoffrey L. Johnston, Neekesh V. Dharia, Fabio L. Luna, Susan McCormack, David Plouffe, Case W. McNamara, John R. Walker, David A. Fidock, Eros Lazzerini Denchi, Elizabeth A. Winzeler

**Affiliations:** 1 Department of Pediatrics, School of Medicine, University of California San Diego, La Jolla, California, United States of America; 2 Biomedical Sciences Program, University of California San Diego, La Jolla, California, United States of America; 3 Department of Microbiology and Immunology, Columbia University College of Physicians and Surgeons, New York, New York, United States of America; 4 School of International and Public Affairs, Columbia University, New York, New York, United States of America; 5 Genomics Institute of the Novartis Research Foundation, San Diego, California, United States of America; 6 Division of Infectious Diseases, Department of Medicine, Columbia University College of Physicians and Surgeons, New York, New York, United States of America; 7 Department of Genetics, The Scripps Research Institute, La Jolla, California, United States of America; University of Washington, United States of America

## Abstract

Malaria parasites elude eradication attempts both within the human host and across nations. At the individual level, parasites evade the host immune responses through antigenic variation. At the global level, parasites escape drug pressure through single nucleotide variants and gene copy amplification events conferring drug resistance. Despite their importance to global health, the rates at which these genomic alterations emerge have not been determined. We studied the complete genomes of different *Plasmodium falciparum* clones that had been propagated asexually over one year in the presence and absence of drug pressure. A combination of whole-genome microarray analysis and next-generation deep resequencing (totaling 14 terabases) revealed a stable core genome with only 38 novel single nucleotide variants appearing in seventeen evolved clones (avg. 5.4 per clone). In clones exposed to atovaquone, we found *cytochrome b* mutations as well as an amplification event encompassing the *P. falciparum* multidrug resistance associated protein (*mrp1*) on chromosome 1. We observed 18 large-scale (>1 kb on average) deletions of telomere-proximal regions encoding multigene families, involved in immune evasion (9.5×10^−6^ structural variants per base pair per generation). Six of these deletions were associated with chromosomal crossovers generated during mitosis. We found only minor differences in rates between genetically distinct strains and between parasites cultured in the presence or absence of drug. Using these derived mutation rates for *P. falciparum* (1.0–9.7×10^−9^ mutations per base pair per generation), we can now model the frequency at which drug or immune resistance alleles will emerge under a well-defined set of assumptions. Further, the detection of mitotic recombination events in *var* gene families illustrates how multigene families can arise and change over time in *P. falciparum*. These results will help improve our understanding of how *P. falciparum* evolves to evade control efforts within both the individual hosts and large populations.

## Author Summary

Malaria is one of the six diseases that together are responsible for 90% of all infectious disease deaths throughout the world. The five species of *Plasmodium* that cause human malaria take over 655,000 lives each year. Parasites evade the immune response through antigenic variation and develop resistance to anti-malarial drugs through genetic changes in either the drug target or genes conferring resistance. We used whole-genome sequencing and microarray techniques to study evolution in *P. falciparum* parasites propagated *in vitro* for up to 180 generations. We determined the mutation rate and found that the core genome of a single clone is stable, while the subtelomeric regions are prone to acquire structural variants. These changes occur mainly in multigene families involved in immune evasion. Our findings indicate that the parasite specifically increases the sequence variability in multigene families through mitotic recombination. This high plasticity of the parasite genome suggests that multiple haplotypes will be present in a natural infection initiated by a single parasite.

## Introduction

Although the global burden of malaria has declined over the last few years to 216 million cases and 655,000 deaths in 2010 [1], the overall goal of global eradication is still out of reach. Emerging resistance to artemisinin, a frontline chemotherapeutic for which resistance is not widespread, has recently been reported along the Thai-Cambodia border (reviewed in [2]). Furthermore, RTS,S, the most advanced vaccine candidate in development, is only minimally effective and does not induce long-lived sterile immunity [3].

A primary reason why malaria is difficult to control is its genome's ability to recombine and/or mutate away from a protective immune response or drug pressure. For example, the development of an effective vaccine has been hampered by the prevalence of strain-specific immunity, where vaccination with one antigenic haplotype protects for only one specific variant [4]. To date, this has been attributed to pre-existing genetic diversity; however, it may also be that escape mutants emerge in vaccinated individuals. Plasticity of the *Plasmodium* genome can also contribute to the evolution of resistance against anti-malarial drugs. Single nucleotide variants (SNVs) and copy number variants (CNVs) in target and resistance genes allow the parasites to evade drug pressure. Most notably, the emergence of chloroquine-resistant parasites ultimately caused a huge resurgence in the number of malaria cases in the 1990s. Although these two mechanisms are well described, it is not understood how often variation arises during mitotic asexual growth or how quickly SNVs accumulate in the absence of selection pressure.

In addition to diversity at the population level, there is also variability within the individual parasite. Multigene families, where only one or few members are expressed, provide antigenetic diversity and allow the parasite to persist in a host. Recombination events which occur in meiosis [5], [6] as well as mitosis [7] give rise to new variants in these already diverse families. This genetic variability in parasites, both in an individual host and on a population level, allows the parasite to evade the host immune system even in the absence of transmission (i.e. during dry seasons).

Given this remarkable genetic diversity, it is not surprising that naturally-infected patients often carry multiple, genetically distinct parasite clones. The multiplicity of infection (MOI) has traditionally been estimated with a handful of genetic markers, which may encode proteins under strong selection by the host immune system. However, these methods are not comprehensive enough to measure true parasite heterogeneity and many variants are missed within an individual [8]–[10]. It is unclear whether parasite heterogeneity is created through multiple infectious mosquito bites, heterogeneity in a single mosquito inoculation, or evolution of new genetic changes (SNVs and structural variants) within the human host.

Knowing the rate at which genetic changes occur is critical to understanding the emergence of drug resistance, the evolution of antigen polymorphisms and multigene families, and the patterns of malaria transmission. It is not possible to study neutral parasite mutation rates in humans due to the influence of selection pressure of the host immune response and genetic host-to-host variability. Previous quantifications of the mutation rate have focused only on single genes under drug selection [11]. The basal rate at which mutations drive *Plasmodium* evolution has therefore never been measured at the whole-genome level and much must be inferred.

Using whole-genome sequencing as well as whole-genome tiling arrays, we have determined the rates at which genetic changes occur in clonal parasite populations in the absence of immune and drug pressure *in vitro*. In addition to the accumulation of SNVs in the core genome, we observed major deletions in the subtelomeric regions and identified seven mitotic recombination events. The rates of these events were not changed by the addition of atovaquone, a commonly used anti-malarial drug.

## Results

### Generation of clonal *P. falciparum* lines in long-term *in vitro* culture

**Figure 1:**
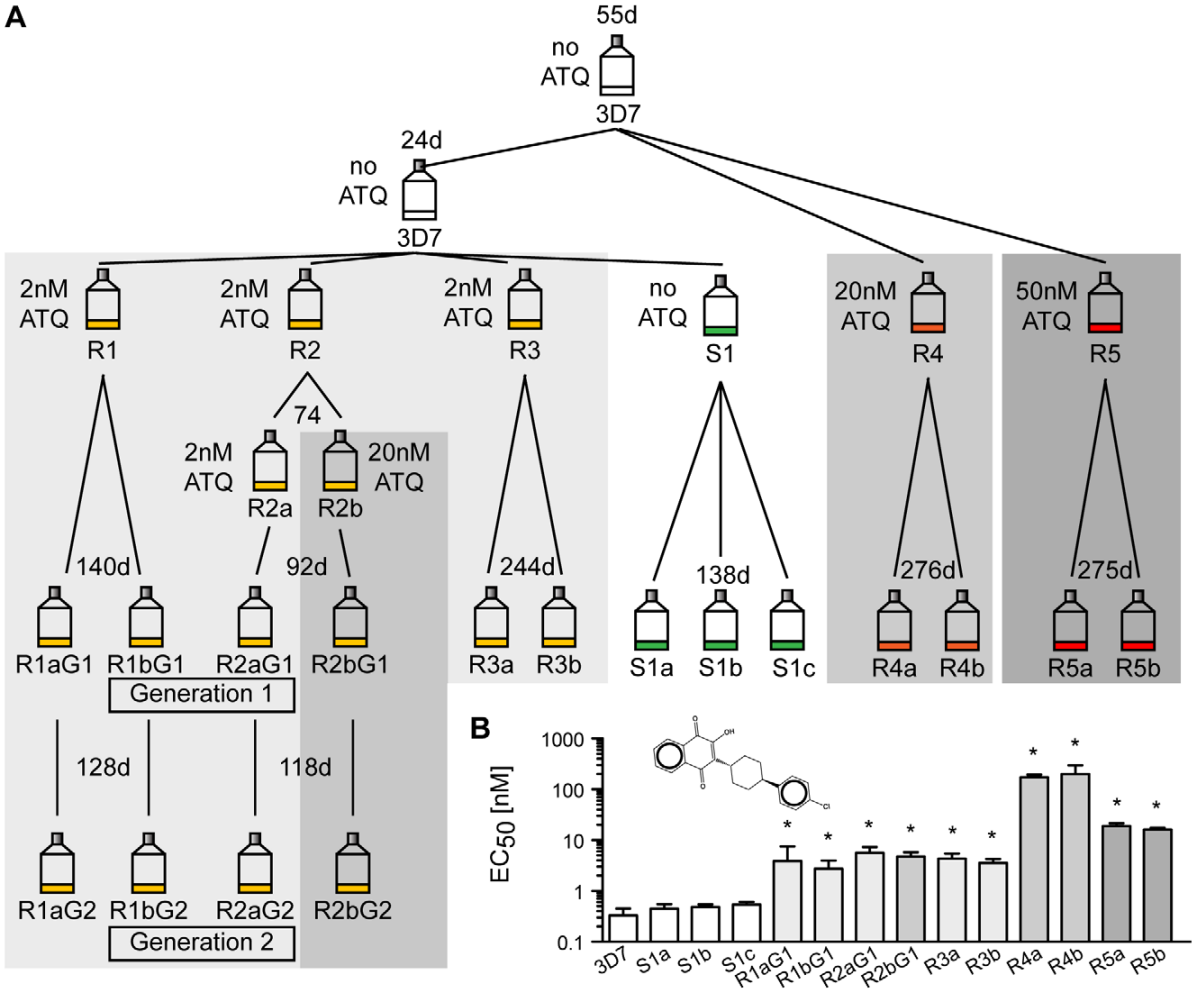
Generation of atovaquone (ATQ)-resistant parasites. A. Selection schematic. An initial parasite clone (3D7) was split into three lines after 55 days, and 20 nM or 50 nM ATQ pressure was applied to lines R4 and R5, respectively. The parental line was kept drug-free. After 24 days, the parental line was split again into four lines, and 2 nM ATQ pressure was applied to three lines (R1, R2, and R3) while line S1 was kept drug free. R2 was again split after 74 days, and 20 nM ATQ was applied repeatedly to line R2b. After the indicated time in culture, all lines were cloned by limiting dilution. Four ATQ-resistant clones were kept in culture (Generation 1 (G1): R1a and b G1 and R2a and b G1) and recloned, resulting in a second generation of clones (Generation 2 (G2): R1a and b G2 and R2a and b G2). The number of days (d) in culture between splits is indicated above each flask. B. ATQ structure and growth inhibition assay. EC_50_ values for 3D7 parent, the sensitive clones, and the ATQ-resistant clones are the means ± SD of three independent experiments performed in quadruplicate. Statistically significant differences between EC_50_ values of the parental 3D7 line and the ATQ-resistant clones were calculated by a one-way ANOVA followed by a Dunnett posttest (*, p<0.0001).

To study the natural genomic plasticity of *P. falciparum*, we investigated how the parasite genome changes over time. A single clonal parent was split into six lines. To investigate the effect of selection pressure on the mutation rate, five parasite lines were exposed to atovaquone (ATQ), a hydroxy-1,4-naphthoquinone that targets the mitochondria-encoded cytochrome *bc* 1 (CYTbc 1) complex of the electron transport chain of *Plasmodium* parasites [12]. ATQ is a component of Malarone, a traveler's medicine drug combination currently used to prevent or treat malaria. Resistance mutations are known to arise quickly [13]. Five lines were exposed to various concentrations of ATQ (R1, R2 and R3: 2 nM, R4: 20 nM, R5: 50 nM) and one line was cultured without drug pressure (S1) for up to a year (Figure 1A). For each line, two clones were selected (three clones for the drug-free line). The four clones of lines R1 and R2 were retained in culture and cloned again (R1a/b and R2a/b, generation 1 and 2). Growth inhibition dose-response assays confirmed that ATQ-resistant clones had indeed acquired at least a 9-fold increase in EC_50_ values for ATQ compared to the 3D7 parent (Figure 1B). Hence, we were able to evolve drug-resistant parasite clones that are 10-fold more tolerant to ATQ than their parent. To facilitate the analysis of different parasites lines on a whole-genome level, all lines were cloned by limiting dilution before being expanded to isolate DNA for further analysis and were as genetically homogenous as could be expected after this process.

### Long-term *in vitro* cultivation of *P. falciparum* reveals few small genomic changes at the whole-genome level

Next we identified the number of genetic changes by comparing the genomes of all seventeen clones to the original 3D7 parental clone using comparative genomic hybridization analysis. The whole-genome tiling microarray we used covers 76% of the coding regions and 41% of the non-coding regions and has a SNV discovery rate of 91% and a false discovery rate of 11% [14]. The PfGenominator software [14] was used to analyze the microarray data and predicted 15 polymorphisms. Capillary sequencing revealed that two of these polymorphisms were deletions, while two were false positives and eliminated from further analysis (Table S1).

As the microarray does not cover the whole genome, the parent and sixteen clones were further analyzed by whole-genome sequencing (WGS) using paired-end 60 bp reads with an average 145 bp insert size. On average, 91.8% of the *P. falciparum* genome was covered by five or more reads, with clones having between 73.5% and 99.9% of the genome covered by fivefold or greater coverage (Table 1). To assess the clonality of our haploid parasite populations, we calculated the number of positions where a significant amount of nucleotides were different from the most prevalent nucleotide. On average, only 235 positions were detected throughout the whole genome and we thus deemed coverage by five or more high quality reads adequate to call SNVs accurately. Areas with less than fivefold coverage included highly repetitive regions such as the telomere repeats and the flanking telomere associated regions (TARs) as well as certain conserved regions within gene families such as the *var*, *rifin*, and *stevor* families. It is therefore possible that some genetic changes in these hard-to-align and poorly annotated regions were not detected.

**Table 1:** Sequencing and microarray results summary.

		Whole-Genome Sequencing (WGS) Statistics			Microarray/WGS Results	
Sample ID	Total days in culture	Total bases [Mb]	Fold genomic coverage	% bases with 5 or more reads	SNVs	Structural variants
3D7 parent	55	1194.5	51.3	99.6	n/a	n/a
R1a G1	219	684.8	29.4	93.5	7	1
R1a G2	347	1825.1	78.3	97.9	10	4
R1b G1	219	1984.7	85.2	98.3	9	2
R1b G2	347	1699.2	72.9	98.7	15	3
R2a G1	245	1108.6	47.6	99.4	3	3
R2a G2	363	1423	61.1	99.4	4	4
R2b G1	245	n/a	n/a	n/a	3	5
R2b G2	363	877.5	37.7	78.3	4	5
R3a	323	931.1	40	76.3	6	6
R3b	323	1116	47.9	75.7	7	4
R4a	331	668	28.7	73.5	6	2
R4b	331	1131.1	48.5	76.9	5	3
R5a	330	1267.7	54.4	97.9	4	3
R5b	330	1309.1	56.2	98.6	4	3
S1a	217	25902.3	1111.7	99.9	3	3
S1b	217	23250	997.9	99.9	1	3
S1c	217	1246.4	53.5	97	1	2
Dd2	45	567.5	24.4	71	n/a	n/a
609_1	236	104.5	4.5	33.8	7	0
609_2	236	145.0	6.2	53.7	5	0
609_3	236	122.7	5.3	42.8	3 (22)*	0
609ctr	236	n/a	n/a	n/a	10	1
678_1	195	237.7	10.2	71	3	1
678_2	195	471.7	20.2	85.4	8 (19)*	1
678_3	195	808.1	34.7	90.3	4	0
678ctr	195	n/a	n/a	n/a	5	0

n/a: not applicable, SNV: single nucleotide variant, Mb: Mega base,

* SNVs in parenthesis were within 15 kb from a chromosome end and could not be confirmed by WGS. ctr: control parasite lines without drug pressure, G1, G2: generation 1 and 2, R: resistant, S: sensitive.

**Figure 2:**
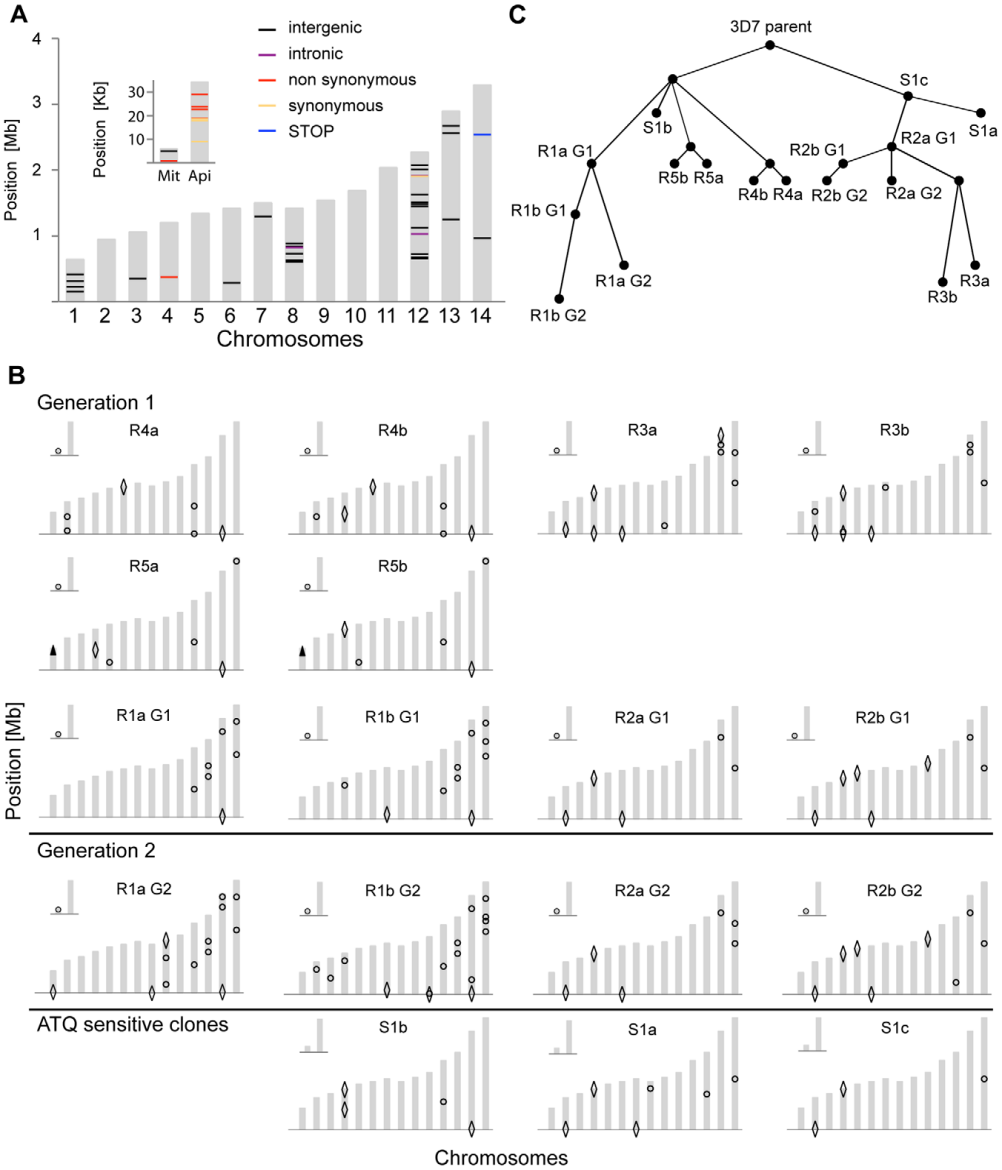
Location and evolutionary relationship of genetic changes following long-term culturing of *P. falciparum* parasites. Genetic changes in each individual clone were detected by microarray analysis as well as by WGS. A. Genetic differences were detected by WGS between our parental 3D7 clone and the available 3D7 reference genome from PlasmoDB v9.1. The 14 chromosomes are indicated in grey, with their relative length on the y-axis. The mitochondrial (Mit) and apicoplast (Api) genomes are shown in the inset. B. Genetic changes identified by microarray and WGS in each individual clone compared to the parental 3D7 clone. SNVs are indicated with circles, large-scale deletions (>1000 bp) with diamonds, and CNVs with arrowheads. For annotations of genes harboring SNVs or that are part of a structural variant, see Table S2. C. Cladogram showing derived evolutionary relationship in B, computed by a heuristic algorithm to find the tree of minimum complexity starting with the parental clone.

The WGS data was analyzed with the PlaTypUS 0.12 software (M. Manary et al., manuscript in preparation), which integrates many community-developed tools into a pipeline to first align the reads to the reference genome and then detect SNVs. To decide on the characteristics of a true SNV, a computer-learning algorithm was trained on a set of 10,500 known SNVs. The WGS data confirmed ten of the fifteen polymorphisms detected by the microarray and identified an additional 25 SNVs across all clones (Figure 2A and Table S2). To verify that a reasonable cutoff was set to call SNVs in PlaTypUS, six SNVs that did not make the cutoff were analyzed by capillary sequencing (Table S1). Four events located next to a poly A or poly T stretch and sequencing confirmed that all clones, including the parent, had the same sequence. The other two events were in fact small deletions but were discarded by the PlaTypUS software as it is designed to detect SNVs only. In summary, 38 SNVs were detected by microarray and WGS (Table S2) and the construction of a cladogram with all polymorphisms in the genome (38 SNVs, nineteen deletions, and one duplication) confirmed the known evolutionary relationship between the clones (Figure 2C).

To estimate how closely related our 3D7 parent is to the reference 3D7 genome, we compared the WGS data to the 3D7 reference sequence from PlasmoDB v9.1. While on average only 5.4 SNVs distinguished a single clone from the parent, we detected 58 SNVs in the parental 3D7 clone relative to the 3D7 reference (Figure 2A and Table S3). It is not clear whether these differences are true SNVs or due to sequencing/assembly errors from the lower coverage reference genome sequencing efforts [15], platform differences, or from the different sources of genomic DNA used in the sequencing projects; however, they highlight the importance of having a recent, isogenic reference genome for such comparative studies.

### ATQ pressure directs the selection but does not alter the mutation rate

**Figure 3:**
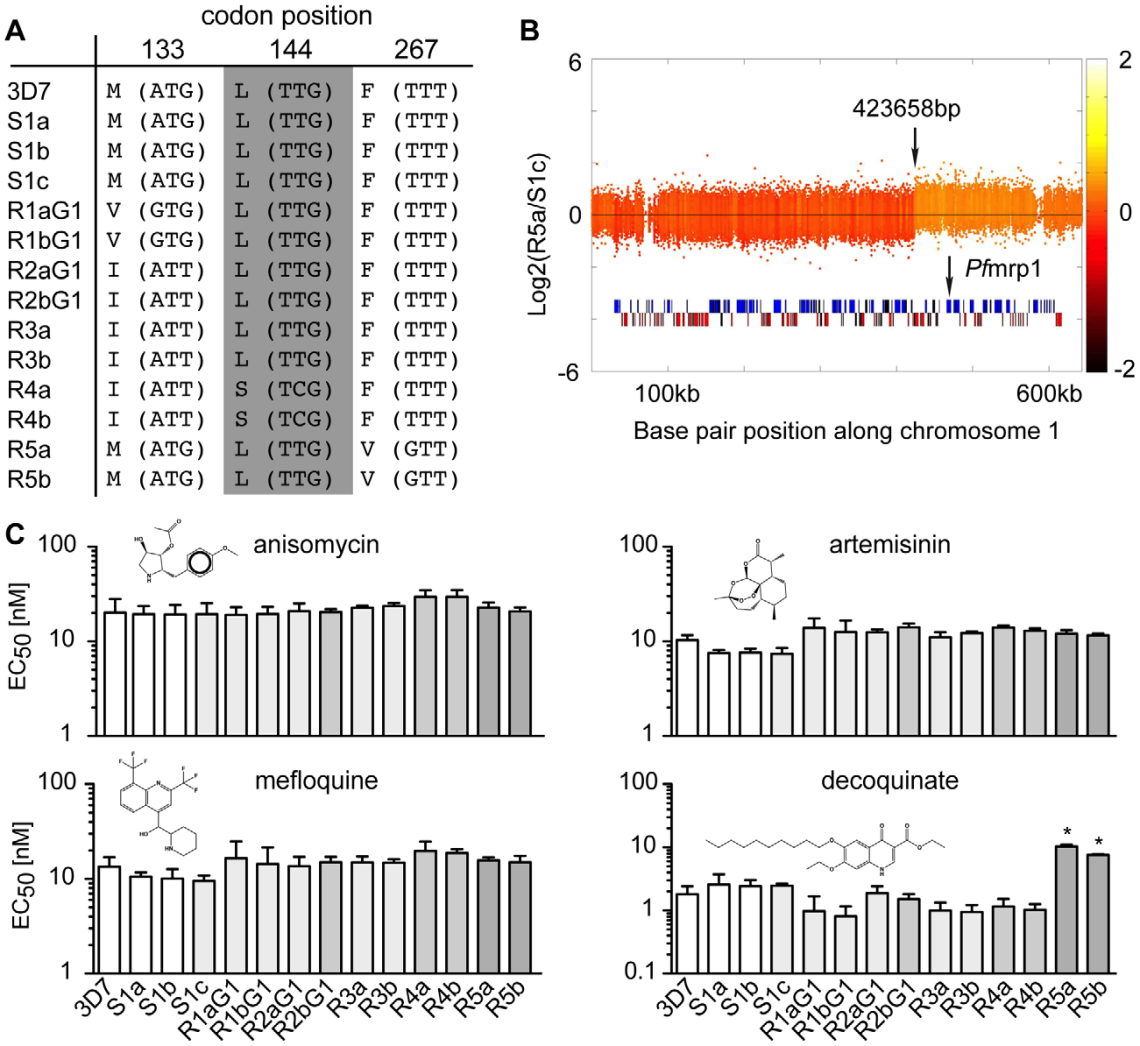
Genomic changes and drug sensitivities of atovaquone (ATQ)-selected clones. A. Amino acid change (nucleotide change) and codon position in cytochrome b of each clone. B. Location of the amplification event detected on chromosome 1 for R5a (423,658–643,292 bp). The location of the multidrug resistance associated protein 1 (*Pf*mrp1, PFA0590w) is shown. The log2 ratio of the intensity of each unique probe in R5a relative to that in S1c is plotted and colored by the moving average over a 500-base pair window. C. The EC_50_ values for four tested drugs are shown for the 3D7 parent, the sensitive clones and the ATQ-resistant clones of the first generation (G1). Statistically significant differences between the EC_50_ values of the parental 3D7 line and the ATQ-resistant clones were calculated by a one-way ANOVA followed by a Dunnett posttest (*, p<0.0001). EC_50_ values are means ± SD of three independent experiments performed in quadruplicate.

We compared the genetic changes acquired in the sensitive clones to the ATQ-resistant clones. As expected, all ATQ-resistant clones acquired SNVs in *cytochrome b*, the target of the drug, while the sensitive clones did not (Figure 2 and Figure 3). Mutations were observed at amino acid position 133 (M133V in R1 pairs and M133I in R2, R3, and R4 pairs), as well as an L144S change in R4 and an F267V mutation in the clones R5. In addition, we found a single amplification event on chromosome 1 in the same R5 sister clones (Figure 3B). Interestingly, this ∼220 kb amplified region encompasses PFA0590w (PF3D7_0112200), which encodes the *P. falciparum* multidrug resistance associated protein 1 (*Pf*MRP1), a protein that has been implicated in parasite resistance to chloroquine and quinine but has not been associated with naphthoquinone resistance [16], [17].

In order to determine if this amplification leads to cross-resistance, we tested our mutants against a series of diverse anti-malarial drugs (Figure 3C). Only the strains that contained the amplification (R5) were cross-resistant to decoquinate, a compound that also targets cytochrome *b* [18] (∼10X, p<0.0001, one way ANOVA followed by a Dunnett posttest). In contrast, the R4 clones, which showed the strongest EC_50_ shift for ATQ compared to the parent, were no more resistant to decoquinate than the parent or the other ATQ-resistant clones. While the presence of ATQ selected for SNVs in *cytochrome b*, it had little influence on the overall number of genetic changes accumulated in ATQ-resistant clones (0.02 genetic changes per days in culture) compared to parasite clones cultured without drug pressure (0.01 genetic changes per day in culture, p = 0.08, unpaired student t test). Our data suggest that external influences such as drug pressure do not appreciably increase the genetic variability of *P. falciparum*.

Having identified the number of isolated genetic changes present in each clone, we calculated the mutation rate per base pair per generation based on the number of mutations detected within each clone and the total time in culture for each clone (Figure 1 and Figure 2) [19], [20]. To accurately calculate the mutation rate, we needed to include not only the observed SNVs but also those SNVs that were lethal/deleterious, which were unobserved due to loss of mutants from the population. The selection force against nonsynonymous mutations causes the appearance of fewer mutants than actually occurred; thus, using observed mutations alone would cause a downward bias in the mutation rate estimate. To correct for this selection bias, we calculated the dN/dS ratio of *P. falciparum* to be 0.59, which confirmed the presence of selection (Materials and Methods). This indicates that 40% (35.5-21)/35.5 of nonsynonymous mutations are deleterious or lethal. Thus, our observed 21 nonsynonymous exonic mutations most likely came from a population of approximately 35.5 true exonic nonsynonymous SNVs. Having accounted for selection, we used a generalized linear model with a linear link function and Poisson distributed error to estimate the mutation rate for each individual clone (Table S2). We calculated an average mutation rate of 1.7×10^−9^ (SD: 1.2×10^−9^) per base pair per generation for the 3D7 lines in the absence of drug pressure and 4.6×10^−9^ (SD: 2.5×10^−9^, student t test alpha >0.05) for the ATQ-resistant clones. These per base pair per generation mutation rates are comparable to other organisms such as yeast (3.3×10^−10^) [21], *Drosophila melanogaster* (8.4 10^−9^) [22], and humans (1.1 to 2.5×10^−8^) [23], [24]. If we assume mutation rates of 1.7×10^−9^ per base pair per generation and a selection disadvantage against nonsynonymous mutations of 40%, this would result in 0.04 mutations per surviving daughter parasite on average. Thus, after 25 generations, every surviving parasite would be expected to have accumulated one mutation relative to its parent.

### Antigenic gene families in subtelomeric regions acquire structural variants

**Figure 4:**
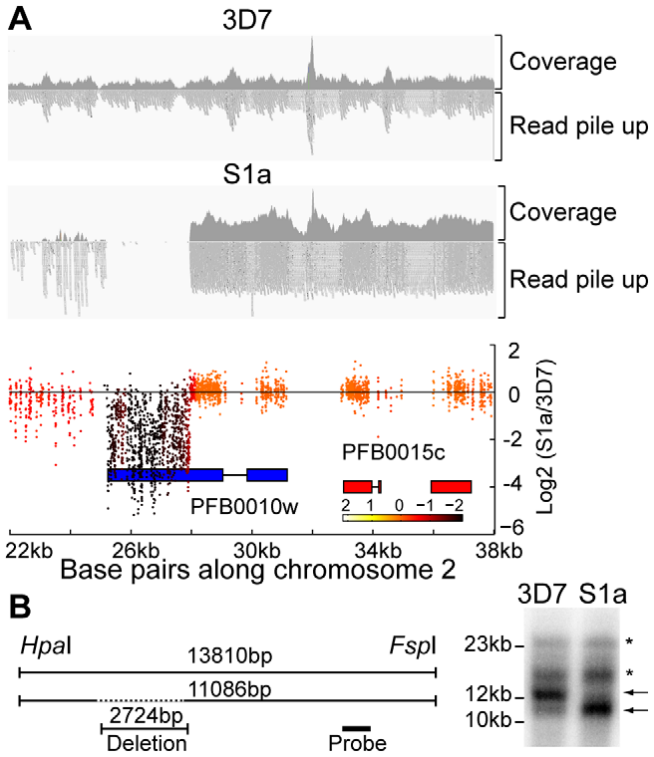
Detection of deletions in subtelomeric regions by WGS and microarray. A. Microarray and WGS detection of deletion events. The top two panels show the number of WGS paired-end reads mapping to 16 kb of chromosome 2 for 3D7 and S1a. The third panel shows the same region but with data from the microarray. The log2 ratio of the intensity of each unique probe for S1a relative to 3D7 parent is indicated and colored by the moving average over a 500-base pair window. B. Southern blot analysis. The gDNA of the 3D7 parent and S1a was cut with restriction enzymes *Hpa*I and *Fsp*I and analyzed by pulsed field gel electrophoresis using a probe to the *rifin* gene PFB0015c adjacent to the *var* gene containing the deletion (schematic on the left, southern blot on the right). Arrows indicate the expected sizes for the fragments of the full-length 3D7 and the truncated S1a *var* gene (PFB0010w). Stars show nonspecific bands.

**Figure 5:**
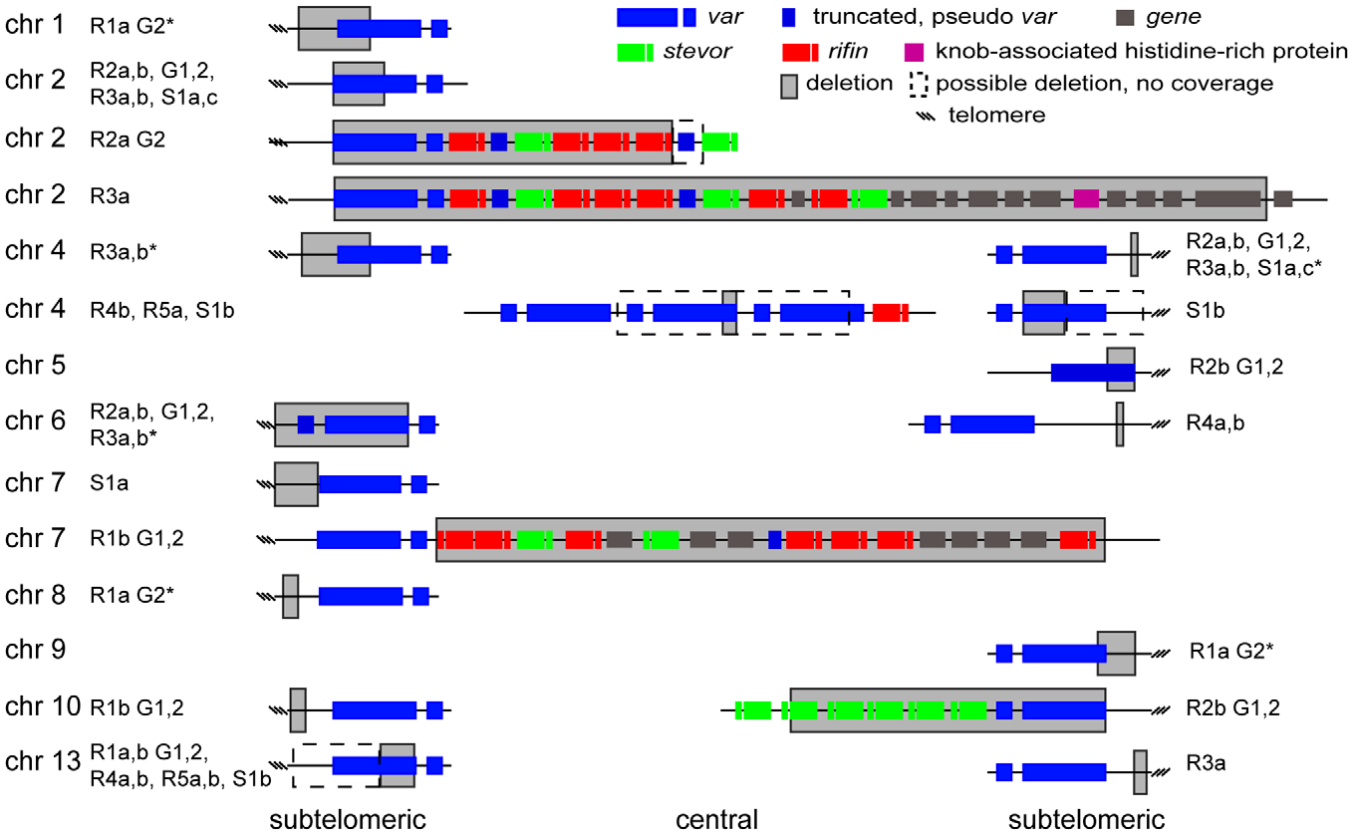
Distribution of deleted genes. Schematic of the location of structural variants detected in individual clones compared to the 3D7 parent. The chromosomal location is indicated on the left, followed by the name of the clone harboring the structural variant (grey box). Dotted boxes indicate regions with low read coverage and absence of unique probes, which mask the exact size of deletions. Different members of gene families are color labeled. Deletions in clones marked with an asterisk are associated with recombination events shown in Figure 6.

Deletions or amplifications of large chromosomal stretches have been observed in long-term *in vitro P. falciparum* cultures as well as in field isolates [10], [25]–[29], with amplifications typically being associated with drug pressure [30]–[32]. We therefore examined the data for structural variants using both microarray (manifested as a substantial loss of hybridization for multiple consecutive probes, or an increase in signal for a block of probes) and WGS (a lack of aligned reads or an increase in read pileup, Figure 4). All derived clones were compared to our parental 3D7 clone. In addition to the above mentioned single amplification event in two sister clones exposed to ATQ (R5a and R5b), we detected eighteen independent large-scale (>1000 bp) deletions of subtelomeric regions (within ∼60 kb of the telomere) and one chromosome internal deletion in the seventeen clones analyzed by microarray as well as WGS (Figure 2, Figure 4, and Figure 5). Comparison of our 3D7 parent to the available 3D7 genome from PlasmoDB v9.1 revealed a deletion on chromosome 2 in our parental clone.

To quantify the appearance of these structural variation events, we calculated a structural variation rate in the same manner as the mutation rates but for the total number of nucleotides deleted or amplified, without the correction for the dN/dS ratio. A structural variation rate of 4.7×10^−6^ per base pair per generation (SD: 1.7×10^−6^) was determined in the absence of drug and 1.0×10^−5^ per base pair per generation (SD: 9.0×10^−6^) in the presence of drug, with the assumption that deletions are fitness neutral. *P. falciparum* subtelomeric regions encode multigene families that are implicated in antigenic variation. Over 80% of the genes involved in structural variation events were members of multigene families with 11 *var*, 14 *rifin*, and 4 *stevor* genes, showing genetic changes that were so substantial that they could no longer be found by WGS or microarray (Figure 5). We confirmed that exon 1 from *var* gene PFB0010c on chromosome 2 was deleted by Southern blot analysis (Figure 4). The identification of deletions at the telomeres confirmed earlier observations from our laboratory where deletions were also common in field isolates [29].

### Deletions are associated with mitotic recombination events

Structural variants can be the result of a variety of events including double-strand breaks and mitotic recombination. To investigate if the observed deletions in multigene families were associated with mitotic recombination events, we further analyzed the boundaries of the detected structural variants in the same paired-end read WGS data set used for variant discovery. Since read pairs are generated on the same fragment of DNA, they normally map to the same chromosome. However, at the edge of a deletion, we found that reads aligning towards the deletion often had read pairs that aligned to a different chromosome, indicating a likely recombination event. The read coverage at the position of the read mate on the other chromosome was often twice the expected number, suggesting a gene conversion event where DNA from one chromosome is added onto another, thereby doubling the donor sequence, while the original sequence in the recipient chromosome is lost.

**Figure 6:**
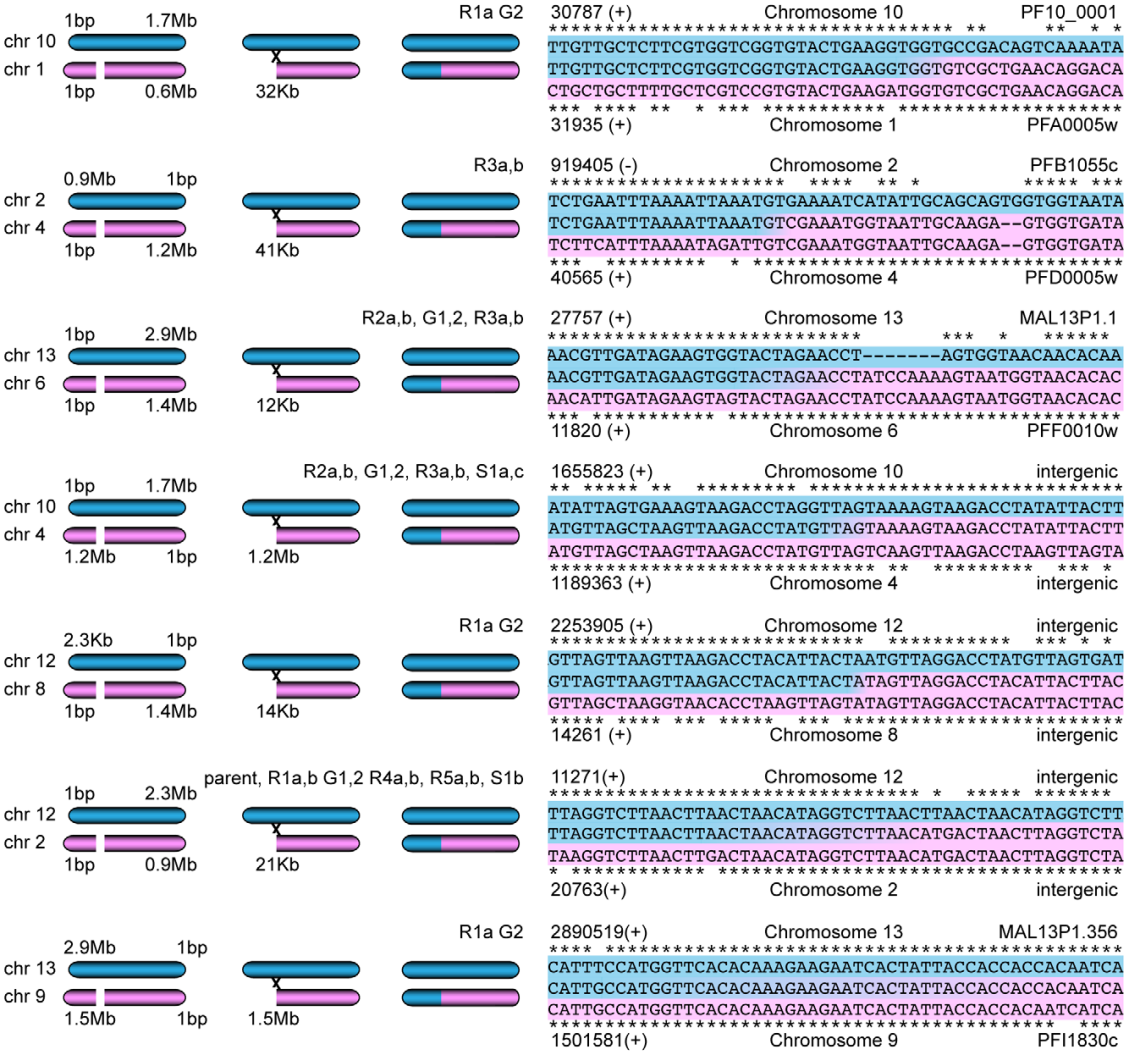
Mitotic recombination events detected by WGS. Paired-end reads next to deletions as well as their read pair mates that mapped to a different chromosome were extracted. *De novo* assembly of these reads, starting with a single seed that mapped next to the deletion, generated new contigs. Hypothetical scenarios of recombination events that created gene conversions are shown on the left; the sequence alignments of the contigs (center sequences), where the two sequences from different chromosomes joined, are on the right. The chromosomal position (with orientation) and the *var* gene ID are indicated when applicable.

To test this hypothesis, we extracted the reads that aligned within 1000 bp of each predicted deletion as well as the reads within 1000 bp of their pair mates on a different chromosome. A read from the deletion site was used as a seed to generate a *de novo* assembly of all these extracted reads. Seven contigs could be assembled that spanned between 1000 and 4000 bp of the sequence next to the deleted region as well as the sequence from a different chromosome indicating a recombination event (Figure 6). Sanger sequencing of three predicted recombination events confirmed the presence of two new chimeric *var* genes consisting of PFF0010w (PF3D7_0600200) and MAL13P1.1 (PF3D7_1300100) as well as PFA0005w (PF3D7_0100100) and PF10_0001 (PF3D7_1000100). The third recombination was already present in the parent and involved intergenic regions close to *var* genes on chromosome 2 and 12.

A previous study also showed gene conversion events between *var* genes from E5 and 3D7, two different clones present in the NF54 isolate; short, 100 bp stretches of a 3D7 *var* gene sequence were found in the context of *var* gene sequences unique to E5 [33]. In contrast, the gene conversions described here are longer and no mosaic sequences were observed. As the parasite is haploid during the asexual blood stage phase of its cycle, the rearrangements of the genome observed here are results of rearrangements during mitotic growth, while the gene conversions described by Frank et al. could be either due to meiotic or mitotic recombination [33].

Not all of the observed deletions were associated with translocation of DNA from one chromosome to another. Broken telomere ends can also be repaired by the addition of telomere repeats to the broken chromosome arm [34], [35]. Therefore, we wanted to investigate if deletions induce a general increase of the telomere repeat length (TRL) on all chromosomes (general increase in the average TRL) or if the telomere repeat size is only increased on broken telomere arms (presence of two populations of TRL). We measured the average TRL by the terminal restriction fragment Southern blot method, where the peak TRL is determined. Of the tested clones, only S1a (containing three deletions) had two peaks indicative of a second population of extended telomere repeats (Figure S1). All other clones had only one peak that was shorter than in the parent, and the average TRL varied between clones from different lines. There was no correlation between the number of deletions and the average TRL of a clone (Figure S1). In addition, clones R1a and R1b from the first generation had longer average TRL than their offspring in generation 2 that had been retained in culture and subcloned again. This indicates that the average TRL fluctuates over time. This also explains the differences observed between the average TRL in this study and an earlier report for 3D7 and Dd2 [34]. These findings show that deletions at the telomeres, in combination with the fluctuation of the average TRL and mitotic recombination events in the asexual erythrocytic cycle can accelerate the evolution of genetic diversity in gene families exposed to the host immune system.

### A stable core genome is common in independently cultured 3D7 clones as well as in a geographically different strain

Based on our findings of genetic variation arising in long-term culture, we predicted substantial changes in the genomes of different circulating clones of 3D7. Three clones from different laboratories were compared to our parental 3D7 by microarray. As expected, all clones contained at least one deletion in the subtelomeric region and only seven small genomic changes in core chromosomal regions were detected in total (Figure S2 and Table S4). Comparing our parental 3D7 to the different 3D7 clones by microarray confirmed a deletion on chromosome 2 in the parent detected by WGS when compared to the reference genome.

To determine if these findings were specific to the 3D7 line, which is susceptible to most drugs, we also analyzed the mutation rate in a *P. falciparum* Dd2 clone and its eight offspring clones that had been selected for resistance to spiroindolones [30]. Dd2 is a multidrug-resistant clone originally derived from W2, a multidrug resistant patient isolate from Southeast Asia, and has been reported to acquire drug resistance at higher rates than other strains (Accelerated Resistance to Multiple Drugs or ARMD [36]). Microarray and WGS analysis detected that the core genome was stable in all Dd2 clones regardless of drug pressure (29 SNVs in six resistant clones and 15 SNVs in two sensitive control clones). Calculation of the mutation rates as described above suggested that Dd2 does not acquire resistance through an intrinsically higher average mutation rate (3.2×10^−9^ and 2.4×10^−9^, in the absence or presence of drug, respectively) (Figure S3 and Table S5), although we cannot rule out that some clones in the W2 strain might have this phenotype. We could detect only one deletion in the eight Dd2 clones that involved a *var* gene, but two clones showed large hybridization differences in subtelomeric regions. The high level of genetic diversity between 3D7 and Dd2 subtelomereric regions makes hybridization analysis with microarray designed for 3D7, or WGS aligned to 3D7, more difficult to interpret; deletions and SNVs could be under- or overestimated in Dd2. Even though the two compounds tested are chemically different and target different pathways, our observations suggest that the mutation rates are similar between different *P. falciparum* strains with different geographical origins.

## Discussion

Using controlled *in vitro* conditions, we were able to reproduce genetic changes (SNVs, deletions in subtelomeric regions and CNVs) usually observed in field isolates and estimate a mutation as well as a structural variation rate for *P. falciparum*. The majority of the 38 SNVs (39%) mapped to intergenic regions or uncharacterized conserved proteins (21%). The appearance of deletions in subtelomeric regions has mainly been attributed to the absence of immune pressure and therefore the lack of counter-selection within *in vitro* cultures. The majority of the genes located in these regions are members of multigene families such as the 60 *var* genes that encode different versions of the *P. falciparum* erythrocyte membrane protein 1 (*Pf*EMP1s). *Var* genes are further sub-grouped into three major groups (A, B and C) based on chromosomal location, orientation and sequence composition of their coding and non-coding upstream regions [37]. Using our new methodology to identify genetic recombination events, we were able to characterize seven recombination events during mitotic growth. Every recombination event observed here occurred either between two group B *var* genes or two group B *var* gene upstream regions, suggesting that recombination events within the same group are more frequent. *Pf*EMP1, an adhesive molecule that is exported to the surface of infected human erythrocytes [38], is a key pathogenicity protein involved in immune evasion. While this adhesive molecule is obsolete in *in vitro* cultures, previous studies showed similar structural variants in the subtelomeric regions of Peruvian field isolates indicating that these deletions are not an artifact of *in vitro* cultures [29].

While we were able to detect structural variants with WGS and microarray analysis, the mechanisms by which they occur remain unclear. The recombination events observed here probably resulted from DNA breaks that were repaired either by direct joining of the broken DNA ends (resulting in a deletion) or by homologous recombination with a closely related *var* gene on a different chromosome (resulting in a gene conversion). While the essential proteins needed for homologous recombination are present in the *P. falciparum* genome, the key determinants for non-homologous end joining (NHEJ) are not [15]. Alternative end joining mechanisms exist in other eukaryotes (reviewed in [39]), though further studies are needed to identify the full repertoire of DNA repair pathways present in *P. falciparum*.

Although we observed deletions in individual clones, it may be that no loss occurs due to DNA translocation to another chromosome in a daughter cell on a population level. Because deletions are much easier to detect than duplications, we might have underestimated the number of the latter. The generation of new chimeric variants of *var* genes could allow the parasite to further evade the immune system, thereby extending its persistence in the patient. This is especially important for the survival of the parasite in semi-endemic areas where there are no meiotic recombination events because no transmission occurs during the dry season. The generation of new variants in polymorphic genes such as the *merozoite surface protein* 1 (*msp*1) [6] or multigene families has largely been attributed to meiotic rather than mitotic recombination. However, our data indicate that ectopic recombination and rearrangement during asexual growth is common between members of antigenic gene families located at the telomeres and could extend to other genes bearing repeated sequence motifs.

An interesting feature of *var* genes is that only one of the several dozen copies is transcribed in any given parasite and transcriptional switching between members occurs [40], [41]. Because humans develop antibodies against this surface-exposed protein, the parasite is believed to use this mutually exclusive expression mechanism to evade the immune response and persist in its human host. *In vitro* switching between members of the *var* gene family has been estimated to occur at a rate of 2% of the parasites per generation in clones derived from IT 4/25/5 [42], [43]. Although this rate is higher than the rate of mitotic change detected here, it may be that genetic change contributes or at least confounds the analysis of *var* gene switching. For example, recombination events and deletions in the subtelomeric regions might interfere with the tethering of chromosome termini to the nuclear periphery [5], [34] or the recruitment of proteins initiating the spread of chromatin, thereby repressing *var* gene transcription (telomere positioning effect) [34], [44], [45]. Differences in average TRL between different strains of *P. falciparum* were reported earlier [34], [35]. We observed alterations in the average TRL of individual clones as well as fluctuation of the average TRL over time, suggesting that the telomere ends are highly dynamic. Evidence that the telomere length could have an influence on the expression of nearby genes was found in Trypanosomes, where the telomere adjacent to the active expression site of VSGs was truncated [46]. All of these events could eventually result in a switch of *var* gene expression. In addition, the changes could further contribute to an evolutionary arms race whereby the host immune response and parasite antigens evolve in parallel to constantly outcompete each other.

The acquisition of new SNVs (most likely due to polymerase errors) and the recombination of the telomeres during mitosis provide the parasite with two mechanisms that generate an enormous amount of genetic diversity. We used a Poisson model to estimate the mutation rate from the number of SNVs accumulated over time in continuous culture (1.7×10^−9^ for 3D7; 4.6×10^−9^ for the ATQ-resistant clones). Our use of the dN/dS-corrected mutation rate from continuous culture provides an estimate that is unaffected by the population bottlenecks caused by dilutions (Materials and Methods). Given that a single human has 10^13^ red blood cells, by the time parasites are visible by thin smear (1% parasitemia) more than 85% of the parasites will have acquired at least one novel genetic change, of which over 53% will be in the repetitive regions of the genome. In chronic infections (more than 30 days), most parasites will differ from one another assuming there is no selection for particular variants. This is supported by a PCR product length analysis of *var* genes in line E5, a clone of a *P. falciparum* patient isolate, NF54 that is also the parental strain of the 3D7 clone. E5 shows at least 13 *var* gene differences to 3D7 [33]. Although we did not detect any differences in major antigens such as *merozoite surface protein 1* or the *circumsporozoite protein*, these genes do bear repeat regions and given that they show high levels of genetic diversity in populations [47], they may also have higher rates of mitotic recombination. These high recombination rates may partially explain why people can be continually re-infected with closely related strains.

While it is accepted in the field that most malaria infections are polyclonal, the actual diversity is still vastly underestimated [9], [10]. The WHO advises the use of only three markers (*merozoite surface proteins 1* and *2*, and *glutamine rich protein*) to establish the MOI in clinical trials on antimalarial drug efficacy [48]. All of the additional genetic changes throughout the parasite genome go unnoticed. Many of these genetic changes might not have an immediate selection advantage to the parasite. However, these unnoticed mutations might become important when a new drug with a new mode of action is introduced and a selection advantage is suddenly introduced. Our results provide the baseline information needed to design diagnostic studies, whole-genome population genetic studies or drug treatment studies.

## Materials and Methods

### Parasites origin and propagation, DNA preparation, and EC_50_ determination

3D7 (MRA-151) was obtained from the Malaria Research and Reference Reagent Resource Center (MR4; American Type Culture Collection deposited by D. Walliker) and cloned in our laboratory at The Scripps Research Institute (TSRI), La Jolla [49]. The San Diego 3D7 clone was derived from the same 3D7 stock as the parental 3D7 propagated at TSRI but was cultured long term at the Genomics Institute of the Novartis Research Foundation before cloning. The St Louis 3D7 clone was obtained from Dan Goldberg and is likely related to MRA-102 (Bei Resources, *Plasmodium falciparum* 3D7, deposited by D. J. Carucci and obtained from D. Walliker) [49], [50] and was used in the genome-sequencing project [51]. The New York strain from David Fidock, Columbia University, was obtained directly from David Walliker. Parasites were propagated in human erythrocytes depleted of white blood cells by filtration using leukocyte filters (Fenwal Inc., Lake Zurich) with medium containing 5% human serum and 1.2% GIBCO AlbuMAX II (Invitrogen, Grand Island) [52]. Parasites were split 1∶20 when they reached parasitemias >8%. To obtain single clones, each line was cloned by limiting dilution (0.25, 0.5, 1, 10 and 30 parasites per well) in 96-well plates and two clones from each line (three clones from the sensitive line) were chosen for further analysis. Clones R1a and b and R2a and b were kept in culture for another 118 (R2a and b) or 128 (R1a and b) days and subcloned again. The original clones were termed generation 1 (R1aG1, R1bG1, R2aG1, and R2bG1) and the offspring were termed generation 2 (R1aG2, R1bG2, R2aG2, and R2bG2). Genomic DNA was purified using a standard phenol-chloroform extraction method [53]. EC_50_ assays were performed using a 384-well format as previously described [54]. Each experiment was repeated three times. The averages of the EC_50_ were calculated for each experiment and log transformed. A one-way ANOVA followed by a Dunnet post-test with the 3D7 parent as reference was performed in Graphpad Prism.

### Microarray analysis and whole-genome sequencing

Microarray analysis was completed following the protocol in Dharia et al. [14]. In brief, fifteen micrograms of genomic DNA and 2.5 ng each of Bio B, Bio C, Bio D, and Cre Affymetrix control plasmids (Affymetrix Inc., Santa Clara) were fragmented with DNaseI and end-labeled with biotin. The samples were hybridized to the microarrays overnight at 45°C in Affymetrix buffers, washed, and then scanned using a modified protocol with wash temperatures of 23°C to account for the high AT content of *P. falciparum*. Briefly, for polymorphism detection we scanned for sets of three overlapping probes that had significantly lower hybridization in the sample when compared to the parental 3D7. Polymorphisms termed SFPs (single feature polymorphism) were detected by z-tests using an empirically derived standard deviation of the normal distribution and a p-value cutoff of 1×10^−8^.

Genomic DNA libraries were prepared for WGS using the standard Illumina protocol of fragmentation, end-polish, and adapter ligation (v. 2011, Illumina, Inc., San Diego). The final PCR enrichment step was conducted with the KAPA HiFi polymerase (KapaBiosystems, Inc., Boston). This enzyme has been shown to more accurately amplify DNA regions with extremely high AT content [55]. DNA libraries were clustered and run on an Illumina Genome Analyzer II, according to manufacturer's instructions. Base calls were made using CASAVA v1.8+ (Illumina, Inc., San Diego).

### Microarray analysis

We have found that different amounts of RNA remaining after the DNA extraction process can give rise to hybridization differences in probes to noncoding RNA (ncRNA) genes. These changes are characterized by hybridization loss or gain over a wide range of probes throughout the gene. Altogether, 12.6% of the 1779 SFPs detected between the parental 3D7 and all clones mapped to ncRNA genes such as tRNAs (15), rRNAs (68), snRNAs (4) the signal recognition particle RNA (3), and MAL8P1.310, which showed similar hybridization patterns and was suspected of encoding an ncRNA. These SFPs were excluded from further analysis.

After excluding these ncRNA genes, we identified a total of 46 SFPs suggestive of SNVs that mapped to 23 different locations in the genome. Blemishes on the microarray surface or nonfunctional probes can give rise to false positives, especially for SNVs with only one or two probes. We assumed that a true SNV would not only be detected between hybridizations of the parent and the clone but also when compared to the other clones. Therefore all clones were also compared to one another to confirm or contradict the initial SFPs detected between the parent and the clone. In the end fifteen SNVs were considered real, of which WGS confirmed ten and capillary sequencing detected two false positives that were excluded from further analysis.

### Whole-genome sequencing analysis

An internally developed WGS pipeline, dubbed PlaTypUS (M. Manary et al., in preparation), was used to align and analyze all WGS data. The program, compiled as a standalone executable, integrates many community-developed tools into its processing pipeline. In order to generate alignments, PlaTypUS follows a multi-step alignment process to generate and execute quality-control measures on an alignment map. FASTQ files obtained from sequencing were aligned to the *Pf*3D7 reference (PlasmoDB v9.1) using BWA v0.6.1 with soft clipping of bases with quality score 2 and below [56]. Those reads that did not map to the reference genome were excluded from further analysis using SAMTOOLS v0.1.18 [57]. PCR duplicates were next identified and removed using Picard v1.48 MarkDuplicates (http://picard.sourceforge.net). Aligned reads were then realigned around indels and areas of high entropy using GATK v1.4+ IndelRealigner. The base quality scores of realigned reads were then recalibrated using GATK TableRecalibration [58]. After realignment and recalibration, the samples were considered ready for use in downstream analysis.

For SNV detection, the PlaTypUS uses a computer-learning algorithm to decide on the characteristics of a true SNV. WGS data for 10,500 known SNVs were obtained from PlasmoDB, and each of these positions was analyzed with regard to 26 metrics. In this way, the profile of a true SNV was discovered. The following criteria were found to be indicative of a true SNV, and were used to filter the set of raw SNVs from our samples. Genetic variants were identified with the GATK UnifiedGenotyper with a minimum base quality score threshold of 20 and subsequently filtered using GATK VariantFiltration with custom filters designed for *Plasmodium* spp [58], [59]. SNVs were excluded if they failed one or more of the following filters: strand bias (p<0.00001, Fisher's exact test), minimum depth of coverage (<5 reads), variant quality (<100 phred scaled), likelihood estimate of genotype being correct (<5 phred scaled), mapping quality bias (p<0.10 using Mann-Whitney Rank Sum test), base quality bias (p<0.10 using Mann-Whitney Rank Sum test), variant quality as a function of depth (<5 per polymorphism), and percentage of non-uniquely mapped reads covering the variant (>10% of total depth with a minimum of 4). Variant annotations to which filters are applied are thoroughly explained on the GATK website (http://www.broadinstitute.org/gsa/). 19,532 raw variant calls across all seventeen clones were filtered down to 38 high quality SNVs. Gene annotations for high quality SNVs were generated using snpEff v.2.0+ (www.snpeff.sourceforge.net).

The PlaTypUS also integrates a novel CNV-calling algorithm, which combines Weierstrass convolution of depth of coverage data with Canny edge detection to identify the break points of copy number events. 18 possible large deletions in the subtelomeric regions were identified for follow-up.

### Determination of mutation rates

We assumed that a number of lethal or deleterious mutations might arise during long-term culture that would not be detected, as the corresponding parasites would be lost; therefore, we tested for evidence of selection by calculating the dN/dS ratio and corrected our observed SNV ratio for this assumed loss. To do this we first calculated the codon potential ratio (the likelihood that a single nucleotide replacement results in a nonsynonymous mutation) from the list of all sequences from the 3D7 reference genome. We utilized the Phylogenetic Analysis by Maximum Likelihood (PAML) software suite [60] (yn00, default settings) to generate a nonsynonymous to synonymous codon potential ratio of 4.63. An uncorrected dN/dS ratio was generated by computing the total number of nonsynonymous SNVs over the length of all possible sites containing those SNVs (the exonic genome) and dividing by the total number of non-nonsynonymous SNVs over their entire domain (the length of the genome). This gave an uncorrected dN/dS ratio of 2.73, which corresponds to a true dN/dS ratio of 0.59, indicating a deficit of nonsynonymous SNVs. In the absence of other information, the assumption was made that the actual ratio should approach 1 in the absence of deleterious mutations, and that only nonsynonymous mutations are deleterious.

We used a generalized linear model with a linear link function and Poisson distributed errors to model the mutation rates for the various lines with and without drug. We assumed that the data followed the model
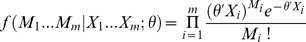
where *M_i_* is the number of mutations scaled by the dN/dS factor; *X_i_* = [*g_i_ l_i_ d_i_*]′ where *g_i_*, is the number of generations in culture, *l_i_*, is a categorical variable indicating the parasite line, *d_i_*, is a categorical variable indicating the presence of drug treatment; *m* is the number of parasite lines; and *θ* is the vector of coefficients to be determined by the model. We used this generalized linear regression to calculate the mutation rate per clone, using the number of SNVs and time to acquire them for each clone separately. We assumed that the mutation rate regression does not include a constant because there are no mutations at baseline. The expected number of mutations per experiment is thus
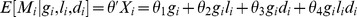



We applied a Poisson-distributed number of mutations with the assumption that each parasite line accumulated mutations with a constant probability per generation. The probability of a mutant occurring in each generation is approximately binomially distributed *B*(*n*,*p*) with *n* the number of nucleotides and *p* the mutation rate. After *g* generations the distribution of the number of mutations per daughter parasite is *B*(*n*,*pg*), assuming replication is independent and identically distributed. These replications are identically distributed because we assume that the accumulation of mutations neither quickens nor slows the mutation rate. By the Poisson theorem, we can approximate *B*(*n*,*pg*) as a Poisson distribution and thus use the regression model as above.

The reason that we can assume a constant mutation rate (i.e. a constant probability of SNVs occurring) is that we have corrected for the selection bias against deleterious mutations using the dN/dS ratio. The dN/dS-corrected number of mutations is an unbiased estimator of the number of mutations that would have occurred in the absence of selection. The number of dilutions of parasite cultures during culture maintenance will not affect this quantity, because we assume that mutations are accumulating in both the remaining and disposed culture at the same constant rate. The number of dilutions *would* affect the mutation rate if we were calculating the rate using the fraction of mutants observed after culturing, because population bottlenecks may increase the variance of the fraction of mutants. Calculating the mutation rate from the proportion of mutants versus wild type organisms after parallel culturing is known as the ‘mutation rate problem’ [20] and the experiments used to calculate these rates are called fluctuation tests. However, we avoid these issues by calculating rates from continuous cultures after correcting for selection pressure [61], [62].

To run these regressions we used the *glm* command (MATLAB R2012b, The Mathworks) and then divided by the number of base pairs in the *P. falciparum* genome (2.35×10^7^) to determine the mutation rates with and without drug.

### Pulsed-field gel electrophoresis and Southern blot

To confirm the partial deletion of PFB0010w on chromosome 2 in clones R2a and b; G1 and 2, R3a and b and S1a and c, 8×10^8^ parasites of the 3D7 parent and the S1a clones were prepared, digested and run on a gel according to previous methods [63], [64]. The chromosome 2 probe was PCR amplified from 3D7 gDNA (chr2_PFB0015cF2: 5′-TACCAACATCGAAAAATACCAAACG-3′ and chr2_PFB0015cR: 5′-TGGCGGAGAGATTTGATGATATTG-3′) and labeled with [^32^P]-αdCTP (3000Ci/mmol). The membrane was incubated with the probe overnight at 42°C in ECL gold hybridization buffer (GE Healthcare) and washed according to the manufacturer's instructions.

To determine the average TRL, mixed-stage parasites cultures of the parental 3D7, all first generation (G1) clones, second generation (G2) clones R1a and R1b, as well as a clonal Dd2 strain were lysed with saponin and the pellets were resuspended in agarose to generate plugs. Agarose plugs containing 8×10^7^ parasites were digested with *Alu*I, *Dde*I, *Mbo*II, *Rsa*I. The digested gDNA was run on an agarose gel and transferred to a membrane. The probe for the telomeres (5′-GGGTTTAGGGTTTAGGGTTTA-3′) was labeled with [^32^P]-γATP and hybridized in Church wash (40 mM NaPi pH 7.2, 1 mM EDTA pH 8, 1% SDS) overnight at 55°C and washed. The membranes were placed against a phosphor storage screen for 2 days and then scanned in a phosphor imager.

The average TRL was estimated by quantifying the signal intensities for each lane using QuantityOne 1-D analysis software (BioRad, Hercules). The area under the curve was then calculated using Prism (GraphPad Software, Inc., La Jolla) and the peak was reported.

### Sanger sequencing

To confirm mutations predicted by microarray and WGS or to confirm accurate rejection of some mutations that did not make the cut off with the PlaTyPus software, 16 predicted SNVs were PCR-amplified with Phusion polymerase (Finnzymes Inc., Woburn) using genomic DNA in a 100 µL PCR reaction volume for 35–40 reaction cycles. Genomic DNA from the 3D7 parent, R1aG2, R2aG2, R3b, R4b and R5b was used as templates. In addition, three predicted gene conversion events were also analyzed by Sanger sequencing. All PCR products were sequenced directly (Retrogen, Inc., San Diego). The primer sequences and results are summarized in Table S1.

### Cladogram analysis

A hierarchical lineage cladogram was constructed from the profile of detected mutations (38 SNVs, 18 deletions, and one duplication) for each clone. Due to the small total number of mutations and the essentially linear process in which they were acquired, an un-weighted pair group method with arithmetic mean was used to generate distances between nodes in the software program Mesquite [65], and a tree diagram was constructed from these calculated distances. Mesquite uses a heuristic algorithm to generate the tree of minimum complexity from the mutation data given as categorical changes (booleans), and then minimizes the number of total number of changes in each tree. Each tree was then re-rooted at the parental strain.

### Reconstruction of recombination events

For each suspected recombination event, a *de novo* assembly of a contig spanning the deletion/recombination event was attempted, using the reads spanning 10,000 bp around the expected site of recombination from both the donor and recipient chromosomes. The PRICE Genome Assembly program (http://derisilab.ucsf.edu/software/price/index.html) was seeded with a single read from the region next to the predicted deletion, whose mate pair aligned to another chromosome, and then run for twenty cycles with otherwise default settings. The largest contig (range 1399 bp to 5405 bp) from each assembly was searched against the entire *Plasmodium* genome using BLAST and was then aligned using ClustalW2 [66] to the two chromosomal regions with the highest identity score and trimmed.

## Supporting Information

Figure S1 Telomere remodeling adds to the natural genetic plasticity of *P. falciparum*. A. Mixed-stage parasite cultures of the parental 3D7, all first generation (G1) clones, second generation (G2) clones R1a and R1b, as well as a clonal Dd2 strain were lysed with saponin and the pellets were resuspended in agarose to generate plugs. Agarose plugs containing 8×10^7^ parasites were digested with restriction enzymes that cut frequently throughout the *Plasmodium* genome except at the telomeres. The digested gDNA was run on an agarose gel and transferred to a membrane. The telomere repeat length (TRLs) were identified using *P. falciparum*-specific radioactively labeled telomere probes. B. The signal intensity for each lane was quantified using QuantityOne. The optical densities (OD) for each position (y-axis in kb) in a lane are plotted on the x-axis. The corresponding average TRLs were calculated for each clone and are indicated in parentheses after the clone's name. The number (#) of deletions of each clone is also indicated in parentheses. C. Relationship between the number of deletions and the average TRL of a clone. The linear regression is indicated. na, not applicable.
(TIF)

Figure S2 Subtelomeric chromosomal differences in independently cultured *P. falciparum* 3D7 clones. The hybridization patterns of clonal 3D7 genomes from different laboratories (Washington University, St Louis [67]; Columbia University, New York [68] and Genomics Institute of the Novartis Research Foundation, San Diego [18]) were compared to our 3D7 (La Jolla) clone's genome pattern. The y axis shows the log2 ratio of hybridization probe intensities for each strain relative to the 3D7 clone used in these experiments, calculated using probes that are unique in the genome and are colored by the moving average over a 500-base pair window as indicated in the color bar. The left panel shows the hybridization pattern for strains without major genetic changes and the right panel, the pattern at the same chromosomal location for a clone from a different laboratory containing a deletion.
(TIF)

Figure S3 Low mutation and deletion rate in Dd2 parasites. A. Schematic of drug selection for Dd2 clones. Starting with a single Dd2 parent, resistant parasite lines were established for two different spiroindolones (NITD609 and NITD678) in triplicate while two lines were cultured in parallel without drug pressure (ctr). B. Clones 678_1 and 678_2 acquired duplications around PFL0590c (labeled by a star), which encodes *Pf*ATP4, the putative target of spiroindolones. Indicated are the log2 ratios of the intensity of each unique probe of the different Dd2 clones relative to those of the Dd2 parent. The probe log ratios were colored by the moving average over a 500-base pair window. C. Chromosomal locations of mutations detected by microarray and WGS (circles) and small deletions (stars).
(TIF)

Table S1 Primers sequences designed for SNV confirmation, cut off verification of PlaTypUS and, gene conversion confirmation. Single nucleotide variants and cutoffs were predicted with PlasmoDB v8.2 and positions and chromosomes are according to PlasmoDB v8.2.
(XLSX)

Table S2 Summary of all deletions and small genetic changes in 3D7 parasites (cultured in the presence or absence of atovaquone) detected by microarray and WGS by an Illumina Genome Analyzer II. PlasmoDB v9.1.
(XLSX)

Table S3 List of all small nucleotide variants detected by the PlaTypUS software between our parental 3D7 clone and the sequence available from PlasmoDB v9.1.
(XLSX)

Table S4 Comparison of 3D7 strains from different laboratories. Gene locations according to PlasmoDB v8.2.
(XLSX)

Table S5 Summary of small genetic changes in Dd2 clones (evolved for spiroindolone resistance) detected by microarray and WGS by an Illumina Genome Analyzer II. Gene locations according to PasmoDB v8.2
(XLSX)

## References

[1] WHO (2011) World Malaria Report 2011. http://www.who.int/malaria/world_malaria_report_2010/en/index.html.

[2] O'Brien C, Henrich PP, Passi N, Fidock DA (2011) Recent clinical and molecular insights into emerging artemisinin resistance in Plasmodium falciparum. Curr Opin Infect Dis 24: 570–577. 22001944 10.1097/QCO.0b013e32834cd3ed PMC3268008

[3] A Phase 3 Trial of RTS,S/AS01 Malaria Vaccine in African Infants. N Engl J Med 10.1056/NEJMoa1208394 PMC10915853 23136909

[4] Genton B, Betuela I, Felger I, Al-Yaman F, Anders RF, et al (2002) A recombinant blood-stage malaria vaccine reduces Plasmodium falciparum density and exerts selective pressure on parasite populations in a phase 1–2b trial in Papua New Guinea. The Journal of Infectious Diseases 185: 820–827. 11920300 10.1086/339342

[5] Freitas-Junior LH, Bottius E, Pirrit LA, Deitsch KW, Scheidig C, et al (2000) Frequent ectopic recombination of virulence factor genes in telomeric chromosome clusters of P. falciparum. Nature 407: 1018–1022. 11069183 10.1038/35039531

[6] Kerr PJ, Ranford-Cartwright LC, Walliker D (1994) Proof of intragenic recombination in Plasmodium falciparum. Mol Biochem Parasitol 66: 241–248. 7808474 10.1016/0166-6851(94)90151-1

[7] Duffy MF, Byrne TJ, Carret C, Ivens A, Brown GV (2009) Ectopic recombination of a malaria var gene during mitosis associated with an altered var switch rate. Journal of molecular biology 389: 453–469. 19389407 10.1016/j.jmb.2009.04.032 PMC3898907

[8] Juliano JJ, Taylor SM, Meshnick SR (2009) Polymerase chain reaction adjustment in antimalarial trials: molecular malarkey? J Infect Dis 200: 5–7. 19469704 10.1086/599379 PMC2803033

[9] Juliano JJ, Porter K, Mwapasa V, Sem R, Rogers WO, et al (2010) Exposing malaria in-host diversity and estimating population diversity by capture-recapture using massively parallel pyrosequencing. Proc Natl Acad Sci U S A 107: 20138–20143. 21041629 10.1073/pnas.1007068107 PMC2993407

[10] Robinson T, Campino SG, Auburn S, Assefa SA, Polley SD, et al (2011) Drug-resistant genotypes and multi-clonality in Plasmodium falciparum analysed by direct genome sequencing from peripheral blood of malaria patients. PLoS ONE 6: e23204 doi:10.1371/journal.pone.0023204. 21853089 10.1371/journal.pone.0023204 PMC3154926

[11] Paget-McNicol S, Saul A (2001) Mutation rates in the dihydrofolate reductase gene of Plasmodium falciparum. Parasitology 122: 497–505. 11393822 10.1017/s0031182001007739

[12] Srivastava IK, Morrisey JM, Darrouzet E, Daldal F, Vaidya AB (1999) Resistance mutations reveal the atovaquone-binding domain of cytochrome b in malaria parasites. Mol Microbiol 33: 704–711. 10447880 10.1046/j.1365-2958.1999.01515.x

[13] Looareesuwan S, Viravan C, Webster HK, Kyle DE, Hutchinson DB, et al (1996) Clinical studies of atovaquone, alone or in combination with other antimalarial drugs, for treatment of acute uncomplicated malaria in Thailand. Am J Trop Med Hyg 54: 62–66. 8651372 10.4269/ajtmh.1996.54.62

[14] Dharia NV, Sidhu AB, Cassera MB, Westenberger SJ, Bopp SE, et al (2009) Use of high-density tiling microarrays to identify mutations globally and elucidate mechanisms of drug resistance in Plasmodium falciparum. Genome Biology 10: R21. 19216790 10.1186/gb-2009-10-2-r21 PMC2688282

[15] Gardner M, Hall N, Fung E, White O, Berriman M, et al (2002) Genome sequence of the human malaria parasite Plasmodium falciparum. Nature 419: 498–511. 12368864 10.1038/nature01097 PMC3836256

[16] Mu J, Ferdig MT, Feng X, Joy DA, Duan J, et al (2003) Multiple transporters associated with malaria parasite responses to chloroquine and quinine. Mol Microbiol 49: 977–989. 12890022 10.1046/j.1365-2958.2003.03627.x

[17] Raj DK, Mu J, Jiang H, Kabat J, Singh S, et al (2009) Disruption of a Plasmodium falciparum multidrug resistance-associated protein (PfMRP) alters its fitness and transport of antimalarial drugs and glutathione. J Biol Chem 284: 7687–7696. 19117944 10.1074/jbc.M806944200 PMC2658063

[18] Nam TG, McNamara CW, Bopp S, Dharia NV, Meister S, et al (2011) A Chemical Genomic Analysis of Decoquinate, a Plasmodium falciparum Cytochrome b Inhibitor. ACS chemical biology 6: 1214–1222. 21866942 10.1021/cb200105d PMC3220786

[19] Foster PL (2006) Methods for determining spontaneous mutation rates. Methods Enzymol 409: 195–213. 16793403 10.1016/S0076-6879(05)09012-9 PMC2041832

[20] Niccum BA, Poteau R, Hamman GE, Varada JC, Dshalalow JH, et al (2012) On an unbiased and consistent estimator for mutation rates. J Theor Biol 300: 360–367. 22326895 10.1016/j.jtbi.2012.01.029

[21] Lynch M, Sung W, Morris K, Coffey N, Landry CR, et al (2008) A genome-wide view of the spectrum of spontaneous mutations in yeast. Proceedings of the National Academy of Sciences of the United States of America 105: 9272–9277. 18583475 10.1073/pnas.0803466105 PMC2453693

[22] Haag-Liautard C, Dorris M, Maside X, Macaskill S, Halligan DL, et al (2007) Direct estimation of per nucleotide and genomic deleterious mutation rates in Drosophila. Nature 445: 82–85. 17203060 10.1038/nature05388

[23] Nachman MW, Crowell SL (2000) Estimate of the mutation rate per nucleotide in humans. Genetics 156: 297–304. 10978293 10.1093/genetics/156.1.297 PMC1461236

[24] Roach JC, Glusman G, Smit AF, Huff CD, Hubley R, et al (2010) Analysis of genetic inheritance in a family quartet by whole-genome sequencing. Science 328: 636–639. 20220176 10.1126/science.1186802 PMC3037280

[25] Pologe LG, Ravetch JV (1988) Large deletions result from breakage and healing of P. falciparum chromosomes. Cell 55: 869–874. 3056622 10.1016/0092-8674(88)90142-0

[26] Biggs BA, Kemp DJ, Brown GV (1989) Subtelomeric chromosome deletions in field isolates of Plasmodium falciparum and their relationship to loss of cytoadherence in vitro. Proc Natl Acad Sci U S A 86: 2428–2432. 2648403 10.1073/pnas.86.7.2428 PMC286926

[27] Patarapotikul J, Langsley G (1988) Chromosome size polymorphism in Plasmodium falciparum can involve deletions of the subtelomeric pPFrep20 sequence. Nucleic Acids Res 16: 4331–4340. 2837730 10.1093/nar/16.10.4331 PMC336633

[28] Shirley MW, Biggs BA, Forsyth KP, Brown HJ, Thompson JK, et al (1990) Chromosome 9 from independent clones and isolates of Plasmodium falciparum undergoes subtelomeric deletions with similar breakpoints in vitro. Molecular and Biochemical Parasitology 40: 137. 1971913 10.1016/0166-6851(90)90087-3

[29] Dharia NV, Plouffe D, Bopp SE, Gonzalez-Paez GE, Lucas C, et al (2010) Genome scanning of Amazonian Plasmodium falciparum shows subtelomeric instability and clindamycin-resistant parasites. Genome Res 20: 1534–1544. 20829224 10.1101/gr.105163.110 PMC2963817

[30] Rottmann M, McNamara C, Yeung BK, Lee MC, Zou B, et al (2010) Spiroindolones, a potent compound class for the treatment of malaria. Science 329: 1175–1180. 20813948 10.1126/science.1193225 PMC3050001

[31] Cowman A, Galatis D, Thompson J (1994) Selection for mefloquine resistance in Plasmodium falciparum is linked to amplification of the pfmdr1 gene and cross-resistance to halofantrine and quinine. Proc Natl Acad Sci USA 91: 1143–1147. 8302844 10.1073/pnas.91.3.1143 PMC521470

[32] Sidhu A, Uhlemann A-C, Valderramos S, Valderramos J-C, Krishna S, et al (2006) Decreasing pfmdr1 copy number in Plasmodium falciparum malaria heightens susceptibility to mefloquine, lumefantrine, halofantrine, quinine, and artemisinin. J Infect Dis 194: 528–535. 16845638 10.1086/507115 PMC2978021

[33] Frank M, Kirkman L, Costantini D, Sanyal S, Lavazec C, et al (2008) Frequent recombination events generate diversity within the multi-copy variant antigen gene families of Plasmodium falciparum. Int J Parasitol 38: 1099–1109. 18395207 10.1016/j.ijpara.2008.01.010 PMC2441941

[34] Figueiredo LM, Freitas-Junior LH, Bottius E, Olivo-Marin JC, Scherf A (2002) A central role for Plasmodium falciparum subtelomeric regions in spatial positioning and telomere length regulation. EMBO J 21: 815. 11847128 10.1093/emboj/21.4.815 PMC125872

[35] Scherf A, Mattei D (1992) Cloning and characterization of chromosome breakpoints of Plasmodium falciparum: breakage and new telomere formation occurs frequently and randomly in subtelomeric genes. Nucleic Acids Res 20: 1491–1496. 1579440 10.1093/nar/20.7.1491 PMC312228

[36] Rathod PK, McErlean T, Lee PC (1997) Variations in frequencies of drug resistance in Plasmodium falciparum. Proceedings of the National Academy of Sciences of the United States of America 94: 9389–9393. 9256492 10.1073/pnas.94.17.9389 PMC23200

[37] Lavstsen T, Salanti A, Jensen AT, Arnot DE, Theander TG (2003) Sub-grouping of Plasmodium falciparum 3D7 var genes based on sequence analysis of coding and non-coding regions. Malar J 2: 27. 14565852 10.1186/1475-2875-2-27 PMC222925

[38] Baruch DI, Pasloske BL, Singh HB, Bi X, Ma XC, et al (1995) Cloning the P. falciparum gene encoding PfEMP1, a malarial variant antigen and adherence receptor on the surface of parasitized human erythrocytes. Cell 82: 77. 7541722 10.1016/0092-8674(95)90054-3

[39] McVey M, Lee SE (2008) MMEJ repair of double-strand breaks (director's cut): deleted sequences and alternative endings. Trends Genet 24: 529–538. 18809224 10.1016/j.tig.2008.08.007 PMC5303623

[40] Scherf A, Hernandez-Rivas R, Buffet P, Bottius E, Benatar C, et al (1998) Antigenic variation in malaria: in situ switching, relaxed and mutually exclusive transcription of var genes during intra-erythrocytic development in Plasmodium falciparum. EMBO J 17: 5418. 9736619 10.1093/emboj/17.18.5418 PMC1170867

[41] Chen Q, Fernandez V, Sundstrom A, Schlichtherle M, Datta S, et al (1998) Developmental selection of var gene expression in Plasmodium falciparum. Nature 394: 392–395. 9690477 10.1038/28660

[42] Roberts DJ, Craig AG, Berendt AR, Pinches R, Nash G, et al (1992) Rapid switching to multiple antigenic and adhesive phenotypes in malaria. Nature 357: 689. 1614515 10.1038/357689a0 PMC3731710

[43] Smith JD, Chitnis CE, Craig AG, Roberts DJ, Hudson-Taylor DE, et al (1995) Switches in expression of Plasmodium falciparum var genes correlate with changes in antigenic and cytoadherent phenotypes of infected erythrocytes. Cell 82: 101. 7606775 10.1016/0092-8674(95)90056-x PMC3730239

[44] Mancio-Silva L, Rojas-Meza AP, Vargas M, Scherf A, Hernandez-Rivas R (2008) Differential association of Orc1 and Sir2 proteins to telomeric domains in Plasmodium falciparum. Journal of cell science 121: 2046–2053. 18525026 10.1242/jcs.026427

[45] Freitas-Junior LH, Hernandez-Rivas R, Ralph SA, Montiel-Condado D, Ruvalcaba-Salazar OK, et al (2005) Telomeric heterochromatin propagation and histone acetylation control mutually exclusive expression of antigenic variation genes in malaria parasites. Cell 121: 25. 15820676 10.1016/j.cell.2005.01.037

[46] Pays E, Laurent M, Delinte K, Van Meirvenne N, Steinert M (1983) Differential size variations between transcriptionally active and inactive telomeres of Trypanosoma brucei. Nucleic acids research 11: 8137–8147. 6324074 10.1093/nar/11.23.8137 PMC326571

[47] Volkman SK, Sabeti PC, DeCaprio D, Neafsey DE, Schaffner SF, et al (2007) A genome-wide map of diversity in Plasmodium falciparum. Nat Genet 39: 113–119. 17159979 10.1038/ng1930

[48] World, Health, Organization (2008) Methods and techniques for clinical trials on antimalarial drug efficacy: Genotyping to Identify Parasite Populations. World Health Organization, Geneva.

[49] Walliker D, Quakyi IA, Wellems TE, McCutchan TF, Szarfman A, et al (1987) Genetic analysis of the human malaria parasite Plasmodium falciparum. Science 236: 1661–1666. 3299700 10.1126/science.3299700

[50] Rosario V (1981) Cloning of naturally occurring mixed infections of malaria parasites. Science 212: 1037–1038. 7015505 10.1126/science.7015505

[51] Gardner MJ, Hall N, Fung E, White O, Berriman M, et al (2002) Genome sequence of the human malaria parasite Plasmodium falciparum. Nature 419: 498. 12368864 10.1038/nature01097 PMC3836256

[52] Trager W, Jenson J (1978) Cultivation of malarial parasites. Nature 273: 621–622. 351412 10.1038/273621a0

[53] Beck HP (2002) Extraction and purification of Plasmodium parasite DNA. Methods Mol Med 72: 159–163. 12125113 10.1385/1-59259-271-6:159

[54] Plouffe D, Brinker A, McNamara C, Henson K, Kato N, et al (2008) In silico activity profiling reveals the mechanism of action of antimalarials discovered in a high-throughput screen. Proc Natl Acad Sci U S A 105: 9059–9064. 18579783 10.1073/pnas.0802982105 PMC2440361

[55] Oyola SO, Otto TD, Gu Y, Maslen G, Manske M, et al (2012) Optimizing Illumina next-generation sequencing library preparation for extremely AT-biased genomes. BMC Genomics 13: 1. 22214261 10.1186/1471-2164-13-1 PMC3312816

[56] Li H, Durbin R (2009) Fast and accurate short read alignment with Burrows-Wheeler transform. Bioinformatics 25: 1754–1760. 19451168 10.1093/bioinformatics/btp324 PMC2705234

[57] Li H, Handsaker B, Wysoker A, Fennell T, Ruan J, et al (2009) The Sequence Alignment/Map format and SAMtools. Bioinformatics 25: 2078–2079. 19505943 10.1093/bioinformatics/btp352 PMC2723002

[58] DePristo MA, Banks E, Poplin R, Garimella KV, Maguire JR, et al (2011) A framework for variation discovery and genotyping using next-generation DNA sequencing data. Nat Genet 43: 491–498. 21478889 10.1038/ng.806 PMC3083463

[59] McKenna A, Hanna M, Banks E, Sivachenko A, Cibulskis K, et al (2010) The Genome Analysis Toolkit: a MapReduce framework for analyzing next-generation DNA sequencing data. Genome Res 20: 1297–1303. 20644199 10.1101/gr.107524.110 PMC2928508

[60] Yang Z, Nielsen R (2000) Estimating synonymous and nonsynonymous substitution rates under realistic evolutionary models. Mol Biol Evol 17: 32–43. 10666704 10.1093/oxfordjournals.molbev.a026236

[61] Nishant KT, Singh ND, Alani E (2009) Genomic mutation rates: what high-throughput methods can tell us. Bioessays 31: 912–920. 19644920 10.1002/bies.200900017 PMC2952423

[62] Rosche WA, Foster PL (2000) Determining mutation rates in bacterial populations. Methods 20: 4–17. 10610800 10.1006/meth.1999.0901 PMC2932672

[63] Hernandez-Rivas R, Scherf A (1997) Separation and mapping of chromosomes of parasitic protozoa. Mem Inst Oswaldo Cruz 92: 815–819. 9566214 10.1590/s0074-02761997000600017

[64] Hinterberg K, Scherf A (1994) PFGE: improved conditions for rapid and high-resolution separation of Plasmodium falciparum chromosomes. Parasitol Today 10: 225. 15275455 10.1016/0169-4758(94)90121-x

[65] Maddison WPaDRM (2011) Mesquite: a modular system for evolutionary analysis. Version 2.75 ed

[66] Larkin MA, Blackshields G, Brown NP, Chenna R, McGettigan PA, et al (2007) Clustal W and Clustal X version 2.0. Bioinformatics 23: 2947–2948. 17846036 10.1093/bioinformatics/btm404

[67] Istvan ES, Dharia NV, Bopp SE, Gluzman I, Winzeler EA, et al (2011) Validation of isoleucine utilization targets in Plasmodium falciparum. Proceedings of the National Academy of Sciences of the United States of America 108: 1627–1632. 21205898 10.1073/pnas.1011560108 PMC3029723

[68] Nkrumah LJ, Muhle RA, Moura PA, Ghosh P, Hatfull GF, et al (2006) Efficient site-specific integration in Plasmodium falciparum chromosomes mediated by mycobacteriophage Bxb1 integrase. Nat Methods 3: 615–621. 16862136 10.1038/nmeth904 PMC2943413

