# Supplementary figures and images for: Mitotic Evolution of Plasmodium falciparum Shows a Stable Core Genome but Recombination in Antigen Families
Source: PLoS Genet. 2013 Feb 7;9(2):e1003293. doi: 10.1371/journal.pgen.1003293 (PMC3567157; doi:10.1371/journal.pgen.1003293)

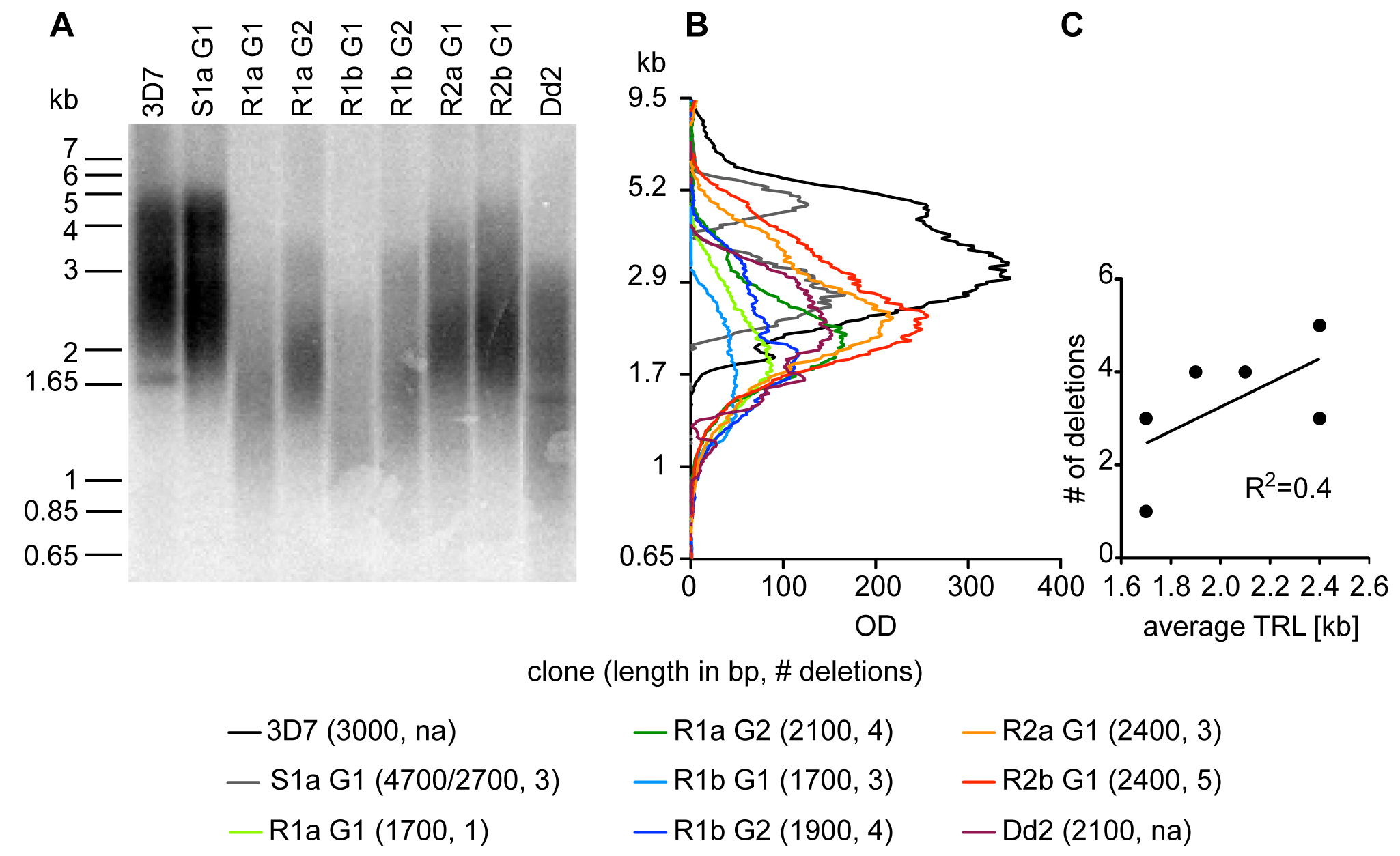

Supplement: Figure S1 — Telomere remodeling adds to the natural genetic plasticity of P. falciparum. A. Mixed-stage parasite cultures of the parental 3D7, all first generation (G1) clones, second generation (G2) clones R1a and R1b, as well as a clonal Dd2 strain were lysed with saponin and the pellets were resuspended in agarose to generate plugs. Agarose plugs containing 8×10^7^ parasites were digested with restriction enzymes that cut frequently throughout the Plasmodium genome except at the telomeres. The digested gDNA was run on an agarose gel and transferred to a membrane. The telomere repeat length (TRLs) were identified using P. falciparum-specific radioactively labeled telomere probes. B. The signal intensity for each lane was quantified using QuantityOne. The optical densities (OD) for each position (y-axis in kb) in a lane are plotted on the x-axis. The corresponding average TRLs were calculated for each clone and are indicated in parentheses after the clone's name. The number (#) of deletions of each clone is also indicated in parentheses. C. Relationship between the number of deletions and the average TRL of a clone. The linear regression is indicated. na, not applicable. (TIF) [file pgen.1003293.s001.tif]

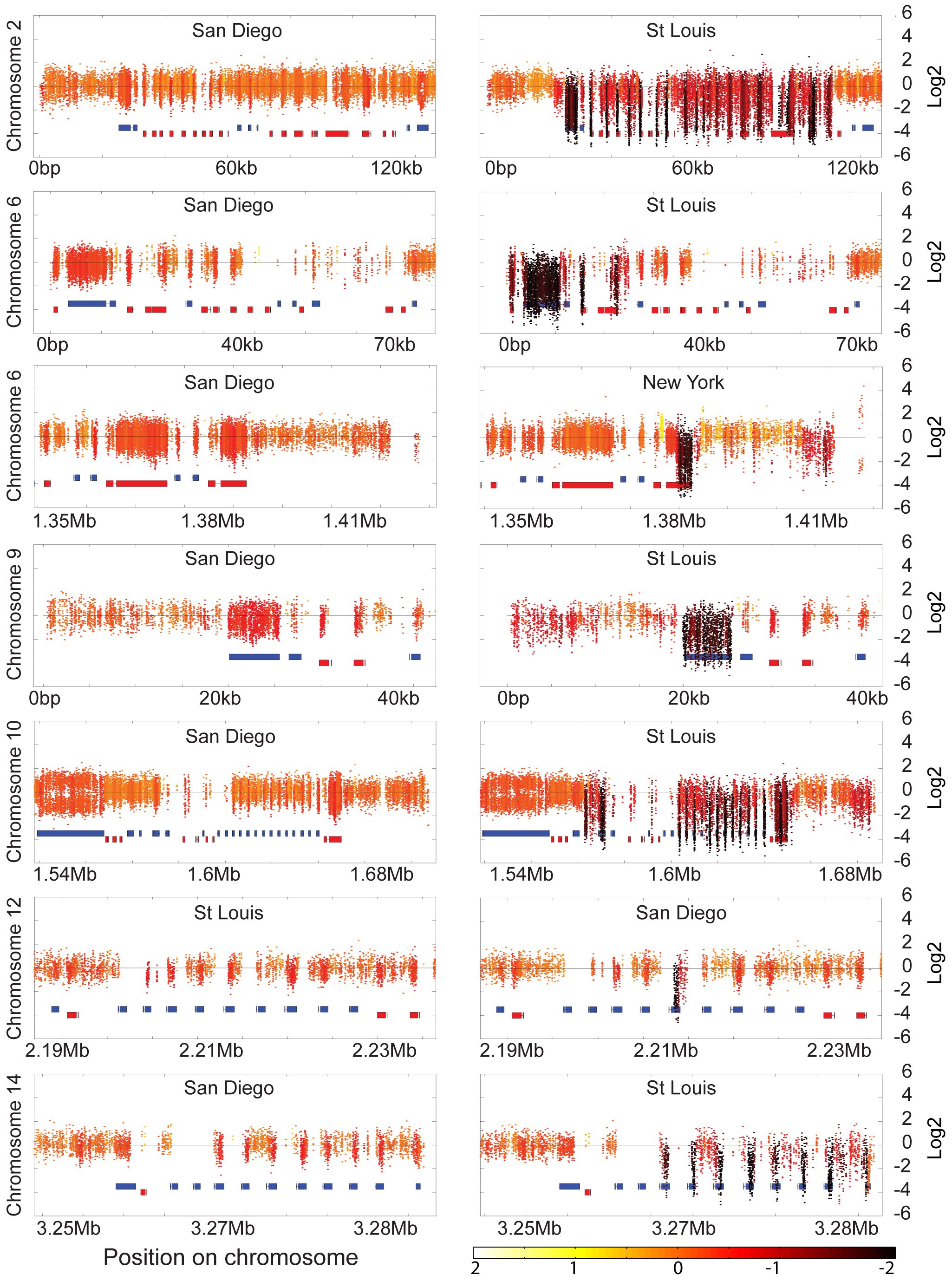

Supplement: Figure S2 — Subtelomeric chromosomal differences in independently cultured P. falciparum 3D7 clones. The hybridization patterns of clonal 3D7 genomes from different laboratories (Washington University, St Louis [67]; Columbia University, New York [68] and Genomics Institute of the Novartis Research Foundation, San Diego [18]) were compared to our 3D7 (La Jolla) clone's genome pattern. The y axis shows the log2 ratio of hybridization probe intensities for each strain relative to the 3D7 clone used in these experiments, calculated using probes that are unique in the genome and are colored by the moving average over a 500-base pair window as indicated in the color bar. The left panel shows the hybridization pattern for strains without major genetic changes and the right panel, the pattern at the same chromosomal location for a clone from a different laboratory containing a deletion. (TIF) [file pgen.1003293.s002.tif]

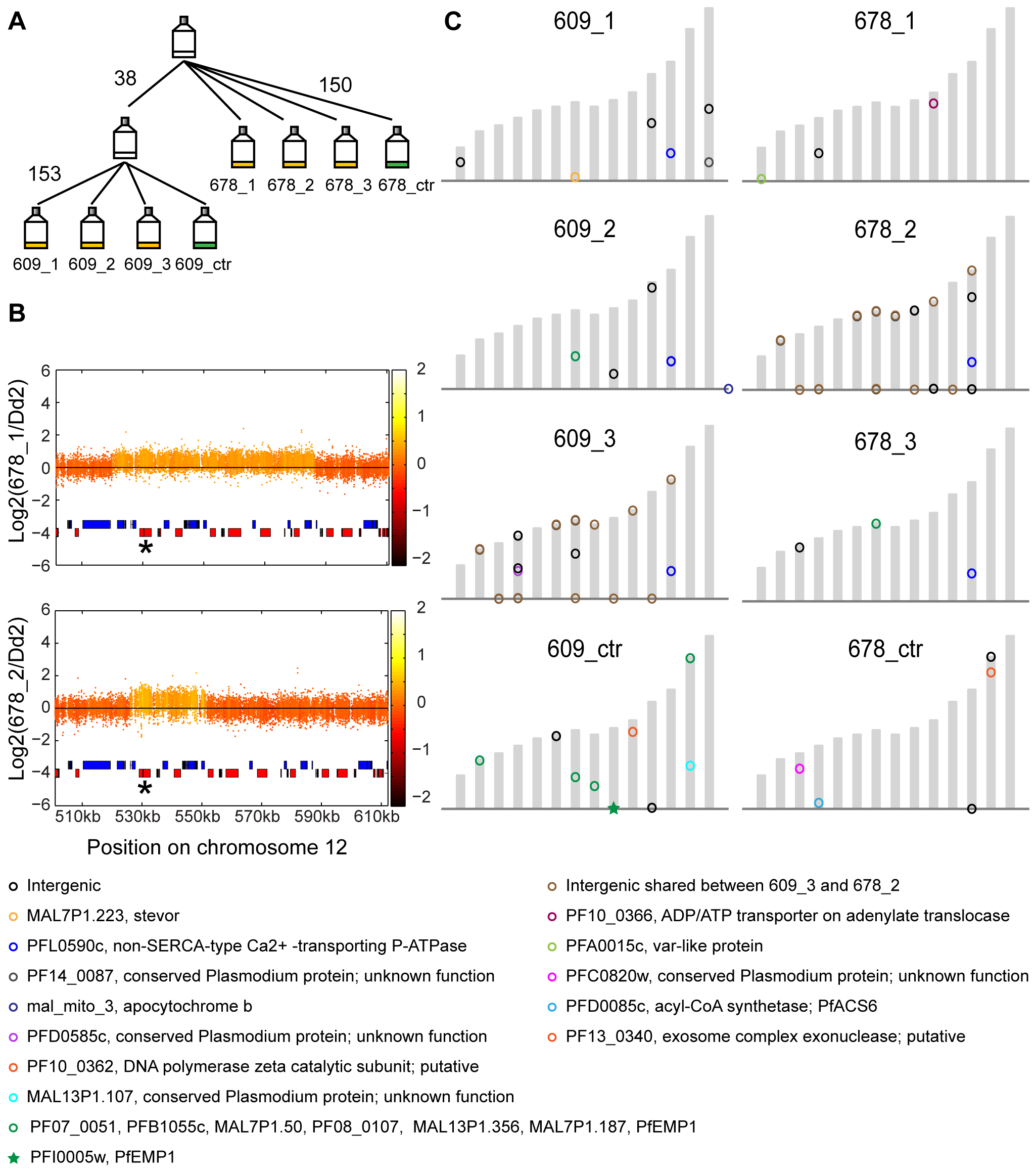

Supplement: Figure S3 — Low mutation and deletion rate in Dd2 parasites. A. Schematic of drug selection for Dd2 clones. Starting with a single Dd2 parent, resistant parasite lines were established for two different spiroindolones (NITD609 and NITD678) in triplicate while two lines were cultured in parallel without drug pressure (ctr). B. Clones 678_1 and 678_2 acquired duplications around PFL0590c (labeled by a star), which encodes PfATP4, the putative target of spiroindolones. Indicated are the log2 ratios of the intensity of each unique probe of the different Dd2 clones relative to those of the Dd2 parent. The probe log ratios were colored by the moving average over a 500-base pair window. C. Chromosomal locations of mutations detected by microarray and WGS (circles) and small deletions (stars). (TIF) [file pgen.1003293.s003.tif]
